# Electrophysiological characterization of granule cells in the dentate gyrus immediately after birth

**DOI:** 10.3389/fncel.2014.00044

**Published:** 2014-02-14

**Authors:** Andrea Pedroni, Do Duc Minh, Antonello Mallamaci, Enrico Cherubini

**Affiliations:** ^1^Department of Neuroscience, Scuola Internazionale Superiore di Studi AvanzatiTrieste, Italy; ^2^European Brain Research InstituteRome, Italy

**Keywords:** dentate gyrus granule cells, immature hippocampus, postnatal development, sodium spikes, low threshold calcium spikes, synchronized network activity, neurogenesis, giant depolarizing potentials

## Abstract

Granule cells (GCs) in the dentate gyrus are generated mainly postnatally. Between embryonic day 10 and 14, neural precursors migrate from the primary dentate matrix to the dentate gyrus where they differentiate into neurons. Neurogenesis reaches a peak at the end of the first postnatal week and it is completed at the end of the first postnatal month. This process continues at a reduced rate throughout life. Interestingly, immediately after birth, GCs exhibit a clear GABAergic phenotype. Only later they integrate the classical glutamatergic trisynaptic hippocampal circuit. Here, whole cell patch clamp recordings, in current clamp mode, were performed from immature GCs, intracellularly loaded with biocytin (in hippocampal slices from P0 to P3 old rats) in order to compare their morphological characteristics with their electrophysiological properties. The vast majority of GCs were very immature with small somata, few dendritic branches terminating with small varicosities and growth cones. In spite of their immaturity their axons reached often the cornu ammonis 3 area. Immature GCs generated, upon membrane depolarization, either rudimentary sodium spikes or more clear overshooting action potentials that fired repetitively. They exhibited also low threshold calcium spikes. In addition, most spiking neurons showed spontaneous synchronized network activity, reminiscent of giant depolarizing potentials (GDPs) generated in the hippocampus by the synergistic action of glutamate and GABA, both depolarizing and excitatory. This early synchronized activity, absent during adult neurogenesis, may play a crucial role in the refinement of local neuronal circuits within the developing dentate gyrus.

## INTRODUCTION

Granule cells (GCs) in the dentate gyrus are crucial for transferring information from the entorhinal cortex to the hippocampus proper where they integrate the classical excitatory trisynaptic circuit (McBain, 2008). Although primarily glutamatergic, the axons of GCs, the mossy fibers (MFs), contain GABA, its synthesizing enzyme glutamic acid decarboxylase (Schwarzer and Sperk, 1995; Sloviter et al., 1996) and the vesicular GABA transporter VIAAT (Zander et al., 2010). In addition, immunogold experiments have demonstrated the presence of both AMPA and GABA_A_ receptors, co-localized on MF terminals in close spatial relation with synaptic vesicles (Bergersen et al., 2003). All these pieces of evidence suggest that MF-cornu ammon (CA3) synapses can use GABA as a neurotransmitter since they posses all the machinery for synthesizing, storing, releasing, and sensing it.

Indeed, electrophysiological experiments from juvenile animals have revealed the presence of mixed GABAergic and glutamatergic monosynaptic currents in CA3 principal cells upon stimulation of GCs in the dentate gyrus (Walker et al., 2001; Gutierrez et al., 2003). Furthermore, in line with the sequential formation of GABAergic and glutamatergic synapses in the immature hippocampus (Hennou et al., 2002), GABA appears to be the only neurotransmitter released from MF terminals during the first few days of postnatal life (Kasyanov et al., 2004; Safiulina et al., 2006, 2010; Sivakumaran et al., 2009) while AMPA/kainate receptor mediated synaptic currents start appearing only after postnatal (P) day 3 (Marchal and Mulle, 2004).

Granule cells are characterized by their peculiar delayed and heterogeneous maturation. Most of them (85%) are generated postnatally. From the primary dentate matrix, neural precursors migrate to the dentate gyrus between embryonic day 10 and 14 where they differentiate into neurons (Altman and Bayer, 1990a,b). Neurogenesis reaches a peak at the end of the first postnatal week and is largely completed toward the end of the first postnatal month (Schlessinger et al., 1975). Interestingly, the dentate gyrus retains the capability to give rise to new neurons throughout life, although at a reduced rate (Duan et al., 2008). In adulthood, after being generated in the subgranular zone, immature GCs are incorporated into pre-existing circuits, thus contributing to improve several brain functions including learning and memory processes (Deng et al., 2010).

The maturation of GCs during postnatal development has been extensively investigated (Liu et al., 1996; Liu et al., 2000; Ye et al., 2000; Ambrogini et al., 2004; Overstreet et al., 2004; Espósito et al., 2005; Overstreet-Wadiche and Westbrook, 2006; Overstreet-Wadiche et al., 2006). However, only few studies, have tried to compare the morphological characteristics of immature GCs with their functional properties before P7, when neurogenesis in the dentate gyrus is very active and GCs exhibit immature-like features (Liu et al., 1996, 2000; Ambrogini et al., 2004).

Therefore, in the present study, whole-cell patch clamp recordings were performed from biocytin-labeled GCs in the immediate postnatal period, between P0 and P3, when GCs convey exclusively monosynaptic GABAergic signals to CA3 pyramidal cells (Safiulina et al., 2006).

## MATERIALS AND METHODS

### ETHICAL APPROVAL

All experiments were performed in accordance with the European Community Council Directive of November 24, 1986 (86/609EEC) and were approved by the local authority veterinary service and by SISSA ethical committee. All efforts were made to minimize animal suffering and to reduce the number of animals used.

### HIPPOCAMPAL SLICES PREPARATION

Wistar rats of both sexes were decapitated after being anesthetized with CO_2_. Hippocampal slices were obtained from neonatal animals at postnatal (P) days P0–P3 (the day 0 was considered as the day of birth) as previously described (Caiati et al., 2010). Briefly, the brain was quickly removed from the skull and placed in ice-cold ACSF containing (in mM): NaCl 130, KCl 3.5, NaH_2_PO_4_ 1.2, MgCl_2_ 1.3, CaCl_2_ 2, Glucose 24, NaHCO_3_ 27 (pH 7.3), saturated with 95% O_2_ and 5% CO_2_ (pH 7.3–7.4)

Transverse hippocampal slices (400 μm thick) were cut with a vibratome and stored at room temperature (20–24°C) in a holding bath containing the same solution as above. After a recovery period of at least 1 h, an individual slice was transferred to the recording chamber where it was continuously superfused with oxygenated ACSF at 31–33°C at the rate of 3–4 ml min^-^^1^.

### ELECTROPHYSIOLOGICAL RECORDINGS

Whole-cell patch clamp recordings (mainly in current clamp mode) were obtained from visually identified GCs in the dentate gyrus, using the Multiclamp 700A amplifier (Axon Instrument, USA).

Patched electrodes were pulled from borosilicate glass capillaries (Hingelberg, Malsfeld, Germany). They had a resistance of 5–8 MΩ when filled with an intracellular solution containing (in mM): KCl 140, MgCl_2_ 1, EGTA 0.5, HEPES 10, Mg ATP 4 (pH 7.3; the osmolarity was adjusted to 280 mOsmol).

The stability of the patch was checked by repetitively monitoring the input and series resistance during the experiment. Cells exhibiting > 15 changes in series resistance were excluded from the analysis. The series resistance was <20 MΩ and was not compensated.

Spontaneously occurring giant depolarizing potentials (GDPs) were routinely recorded from a holding potential of -70 mV.

### DRUGS

Drugs used were: tetrodotoxin (TTX, purchased from Latoxan, Valence, France), 6,7-dinitroquinoxaline-2,3-dione (DNQX), bicuculline methiodide (purchased from Tocris Cookson Inc., UK), and biocytin (purchased from Sigma-Aldrich Milano, Italy). All drugs were dissolved in ACSF except DNQX that was dissolved in DMSO. The final concentration of DMSO in the bathing solution was 0.1%. At this concentration, DMSO alone did not modify the shape or the kinetics of synaptic currents. Drugs were applied in the bath via a three-way tap system, by changing the superfusion solution to one differing only in its drug(s) content. The ratio of flow rate to bath volume ensured complete exchange within 2 min.

### DATA ACQUISITION AND ANALYSIS

Data were acquired and digitized with an A/D converter (Digidata 1200, Molecular Devices) and stored on a computer hard disk. Acquisition and analysis were performed with Clampfit 9 (Axon Instruments, USA). Data were sampled at 20 kHz and filtered with a cut off frequency of 2 kHz. The resting membrane potential (RMP) was measured immediately after break-in and establishing whole-cell recording. The input resistance (R_in_) was calculated by the slope of the linear portion of the I/V relationship obtained by measuring the steady-state potential changes in response to hyperpolarizing current steps of increasing intensity (from -60 to +120 pA, 20 pA increments, 500 ms duration) using the Clampfit program (pClamp 9.0 software, Axon Instrument, USA). The membrane surface was estimated in voltage clamp mode by integrating the area under the average of four uncompensated and unfiltered charging transients in response to hyperpolarizing steps from a holding potential of -60 mV.

Action potentials were evoked in current clamp mode from a holding potential of -60 mV by 500 ms depolarizing current pulses. Spike width was measured at the base of action potentials and spike amplitude from the baseline to the peak. Spike threshold was determined at the beginning of the fast up rise of an action potential. Possible sag in electrotonic potentials were identified by injecting hyperpolarizing current pulses of different intensities through the recording pipette.

Unless otherwise stated, data are presented as mean ± SEM. Quantitative comparisons were based on students paired or unpaired *t*-test, as required and a *p* value < 0.05 was considered as significant.

### CELL STAINING

*Post hoc* identification of recorded cells was achieved by injecting biocytin (1–2%, from Sigma Aldrich, Milano, Italy, dissolved in the internal solution) throughout the recording electrode for at least 40–60 min. After electrode removal slices were kept in the recording chamber, continuously superfused for at least 15–20 min. to optimize the complete diffusion of biocytin. Slices were then removed from recording chamber, thoroughly washed with phosphate buffered saline (PBS) 1X and fixed with parafolmaldehyde 4% for 20 min. at room temperature and stored at 4°C. Slices were incubated with Alexa Flour 647-labeled streptavidin, 1:500 for 1 h at room temperature, sheltered from the light. They were washed, thoroughly rinsed with 1x PBS, mounted onto slides, embedded with Vectashield (Vector Laboratories), and coverslipped. Individual pictures of biocytin-filled cells were acquired with a Nikon microscope (Eclipse Series TiE, equipped with a C1 confocal system) along progressive focal planes to fully cover their volume (including their dendritic and axonal projections).

### IMMUNOCYTOCHEMISTRY

Free-floating recorded slices were rinsed several times with 1x PBS and incubated in a blocking solution containing 5% FBS (fetal bovine serum) and 0.3% Triton X-100 in PBS, for 30 min. Primary antibodies (anti-Prox1, ab37128, rabbit polyclonal, Abcam, Cambridge, MA, USA 1:500; anti-NeuN, MAB377, mouse monoclonal, Millipore, Billerica, MA, USA, 1:500), diluted in 95% PBS-5% FBS solution, were applied and incubated at room temperature for 2 h. Then, slices were washed several times with 1x PBS and incubated with secondary antibody (Alexa 488-conjugated goat anti-mouse immunoglobulin G [IgG], 1:500, 594-conjugated goat anti-rabbit IgG, 1:500, Alexa Flour 647-labeled streptavidin, 1:500 and 4,6′-diamidino-2-phenylindole [DAPI], 1:1000) for 1 h at room temperature, sheltered from the light. Finally, slices were washed, thoroughly rinsed with 1x PBS, mounted onto slides, embedded with Vectashield (Vector Laboratories), and coverslipped.

## RESULTS

### IDENTIFICATION OF GRANULE CELLS

**FIGURE 1:**
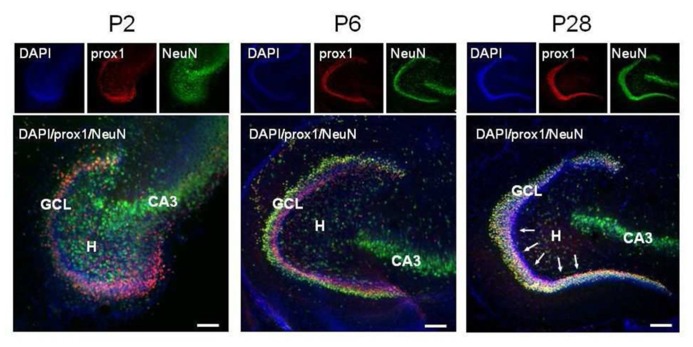
**Representative confocal images of immunostained P2, P6, and P28 horizontal sections of the hippocampus.** DAPI staining, Prox1 and NeuN immunoreactivity are in blue, red, and green, respectively. Pink (DAPI+, Prox1+, NeuN-), immature granule cells predominate in the coalescing P2 GCL, they occupy the inner half of the P6 GCL and are rare in the P28 GCL, where they are confined to a row close to the subgranular zone (arrows). GCL, granule cell layer; H, hilus; CA3, cornu ammonis 3 field.

Granule cells were identified thanks to their immunoreactivity for Prox1. This is a homeoprotein expressed in several brain regions including the dentate gyrus, where it is present throughout development and in adulthood (Lavado and Oliver, 2007). Mature GCs were further distinguished as immunoreactive for NeuN. This is a nuclear antigen expressed in most neuronal cell types throughout the adult nervous system (Mullen et al., 1992), which is specifically activated in GCs by the end of their maturation (Ming and Song, 2011; Hsieh, 2012; Iwano et al., 2012). The spatio-temporal distribution of immature GCs in the dentate area was investigated at three postnatal stages: P2, P6, and P28. As shown in **Figure 1**, at P2, NeuN-positive cells were mainly clustered in the pyramidal layer of the CA3 region and scattered throughout the hilus. At this age, only a few NeuN-positive cells where found in the coalescing Prox1-positive dentate gyrus, where NeuN co-localized with Prox1. At P6, comparable numbers of Prox1-positive/NeuN-negative and Prox1-positive /NeuN-positive cells were detectable within the inner layer and the outer layer of the granule cell layer (GCL), respectively, Finally, at P28, almost all Prox1-positive neurons expressed NeuN, except a few NeuN- elements close to the subgranular zone. In a few words, immature GCs, largely prominent at P2, coexist with similar numbers of mature elements at P6 and become a minority by P28.

### BIOCYTIN-LABELED GCs EXHIBIT AN IMMATURE PHENOTYPE

**FIGURE 2:**
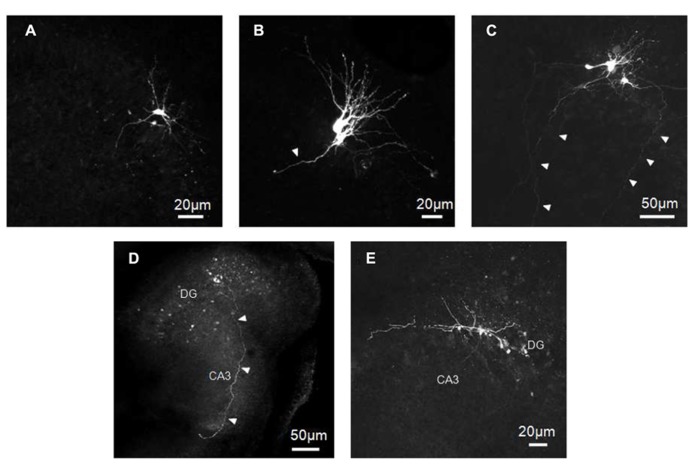
**Biocytin-labeled GCs at P0–P2.** GCs exhibit small cell bodies with few short and tick dendrites oriented toward the molecular layer **(A–C)** and/or running tangentially to the GC layer **(C,E)**. Dendrites often display small varicosities, filopodia and growth cones **(B,C,E)**. Presumed GC axons projecting through the hilus toward stratum lucidum can be seen in **C,D**. Arrowheads indicate presumable axons.

Immunocytochemical data have clearly demonstrated that at P2 GCs exhibit a typical immature phenotype. To fully characterize the functional properties of these cells, stable whole-cell recordings (mainly in current clamp configuration), lasting more than 30 min, were obtained from 63 putative GCs in slices obtained from P0 to P3 old rats. Some of these cells (11/63), were intracellularly labeled with biocytin. Cells were identified as GCs on the basis of their cell bodies localized in the GC layer and dendrites oriented toward the molecular layer. The vast majority of labeled cells exhibited small bodies and few short dendrites emerging mainly from the top or sides of cell bodies, oriented toward the molecular layer or running tangentially to the GC layer (**Figure 2**). In comparison with more mature GCs (see Liu et al., 2000; Overstreet et al., 2004; Markwardt et al., 2009) dendrites never penetrated deeply into the molecular layer or reached the top (**Figures 2A–C**). They were short, thick and spineless with limited branching. They often displayed small varicosities, filopodia and growth cones (**Figures 2A–C,E**). Presumed GC axons with initial extension toward the hilus could be visualized. In four cases, these could be followed up to stratum lucidum in the CA3 subfield (**Figures 2C,D**). The axons expressed varicosities but lacked mature MF boutons and often gave rise to collateral branches that terminated with growth cones. Although care was used to pull out the patch pipette from the recorded neuron at the end of the experiments, more than one GC was often labeled, suggesting dye-coupling (**Figures 2** and **7A**). However, due to their small diameter, we failed to patch two adjacent neurons to verify whether dye-coupled cells were also electrically coupled.

### ELECTROPHYSIOLOGICAL CHARACTERISTICS OF IMMATURE GCs

Immature GCs were identified as neurons by their capacity to generate action potentials. We examined firstly the passive membrane properties (*n* = 63). On average, the RMP was -39 ± 1 mV (ranging from -58 to -23 mV); the input resistance (R_in_) 1.2 ± 0.1 GΩ (ranging from 0.3 to 2.9 GΩ), the membrane capacitance (C) 15.4 ± 0.7 pF (ranging from 8 to 31 pF) and the membrane time constant (τ) 285 ± 17 ms.

**FIGURE 3:**
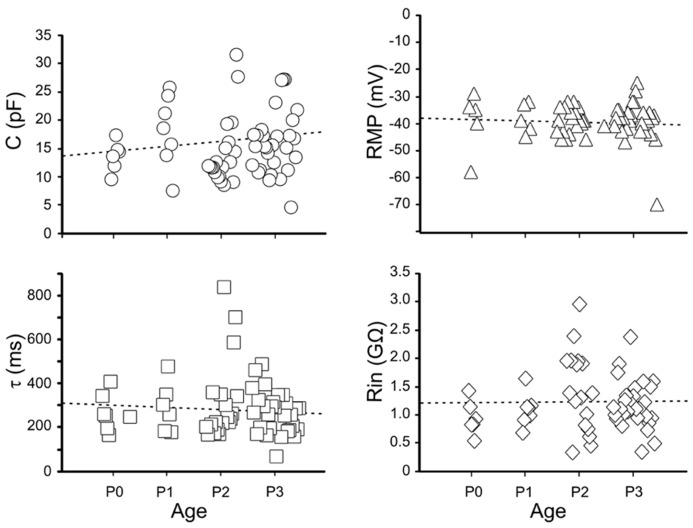
**Passive membrane properties of immature granule cells.** Individual values of capacitance (C, open circles), resting membrane potential (RMP, open triangles), decay time constant (τ, open squares) and membrane input resistance (R_in_, open diamonds), detected between P0 and P3.

A large variability of individual RMP, C, and R_in_ values occurred between P0 and P3 (**Figure 3**). A large variability was also observed within the same postnatal group and between different cells recorded from the same slice. In spite of similar values of RMP, R_in_, capacitance and membrane time constant, immature GCs exhibited marked changes in their excitability as assessed by the large variability in spike detection. Four cells, exhibiting relatively low R_in_ (0.8 ± 1 MΩ), more depolarized RMP (-35 ± 1 mV) and low capacitance values (8 ± 1 pF) were unable to generate action potentials in response to depolarizing currents pulses (non-spiking cells). These cells could be non-differentiated progenitors, astrocytes, oligodendrocytes and/or very immature neurons. Therefore, they were excluded from the present analysis.

Spiking neurons were divided in two groups on the basis of their ability to generate over-shooting action potentials or not (**Table 1**).

**Table 1:** Passive and active membrane properties of granule cells at P0–P3 (*p < 0.05; **p < 0.01).

	**Immature neurons with rudimentary spikes**	**More mature neurons with overshooting action potentials and repetitive firing**
n	36	27
C (pF)	14 ± 1	17 ± 1*
RMP (mV)	-38 ± 1	-40 ± 1
R _ in_ (GΩ)	1.4 ± 0.1	1 ± 0.1**
τ (ms)	283 ± 23	285 ± 25
Spike threshold (mV)	-26 ± 1	-34 ± 1**
Spike amplitude (mV)	13 ± 1	22 ± 2**
Spike half-width (ms)	6.1 ± 0.6	3.8 ± 0.2**

**FIGURE 4:**
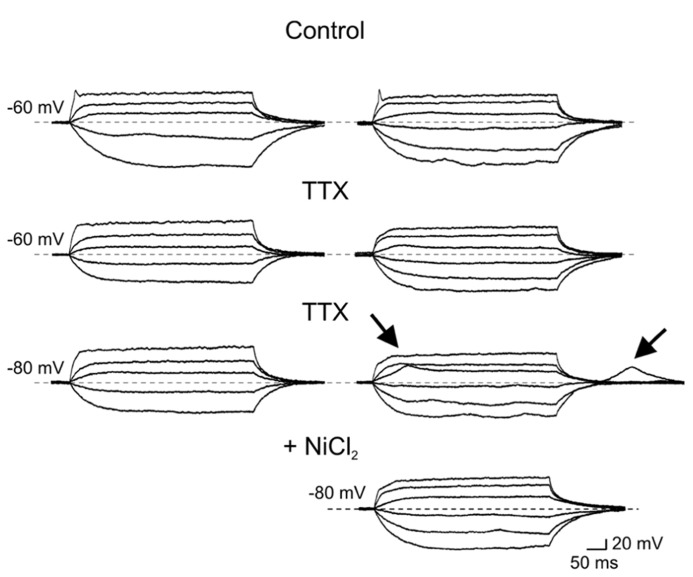
**Immature granule cells exhibit rudimentary sodium spikes and low threshold calcium spikes.** Voltage responses obtained in two GCs (at P2) exhibiting sodium (right and left columns) and low threshold calcium currents (right). In control, both GCs exhibited small amplitude rudimentary spikes (at -60 mV, upper traces) that were blocked by TTX (1 μM). In the presence of TTX, depolarizing current pulses from -80 mV evoked only in the cell to the right a low threshold calcium spike. This was also present upon membrane re-polarization following an hyperpolarizing current step. Low threshold calcium spikes (marked by arrows) were blocked by nickel (100 μM, lower trace on the right).

**FIGURE 5:**
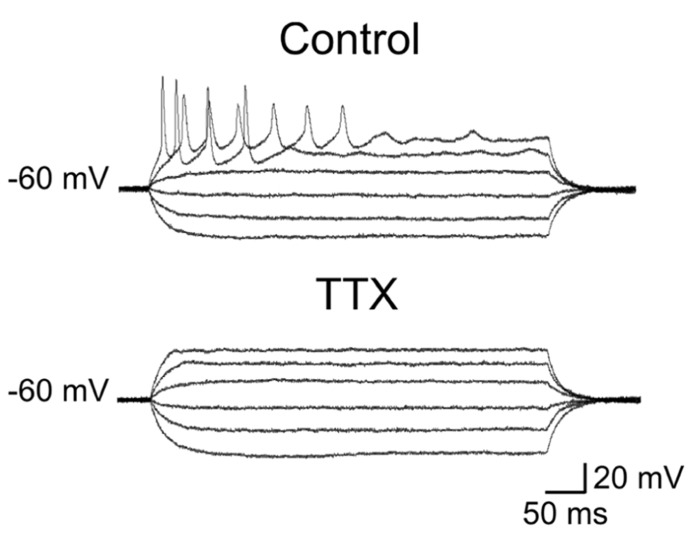
**Repetitive firing evoked in more mature granule cells.** Voltage responses to depolarizing and hyperpolarizing currents steps of increasing intensity. Note clear overshooting spikes firing repetitively during depolarizing current pulses. Action potentials were readily blocked by TTX.

The first group (*n* = 36) comprised more immature cells with rudimentary short and wide TTX-sensitive sodium spikes. Often in the presence of TTX, low threshold calcium spikes appeared and these were blocked by low concentrations of nickel (100 μM; **Figure 4**). The second group of cells (*n* = 27) was characterized by clear overshooting action potentials, which in some cases fired repetitively (**Figure 5**).

In comparison with more immature cells, these exhibited a lower threshold for action potential generation, and a reduced spike half-width value (**Table 1**).

In the presence of TTX, low threshold calcium spike could be sometimes evoked from a more hyperpolarized holding potential (-80 mV; **Figure 4**).

Both groups exhibited electrotonic potentials that strongly rectified in the depolarizing direction, probably due to the activation of voltage-gated potassium conductances. In 23 GCs exhibiting both rudimentary and more mature spikes, a prominent time-dependent sag in the electrotonic potentials could be elicited by hyperpolarizing current steps. The sag accounted for most of the rectification in the hyperpolarizing range and had the characteristics of the time-dependent inward rectifier cationic current I_Q_, described in the hippocampus (Halliwell and Adams, 1982; data not shown).

### CORRELATED NETWORK ACTIVITY

**FIGURE 6:**
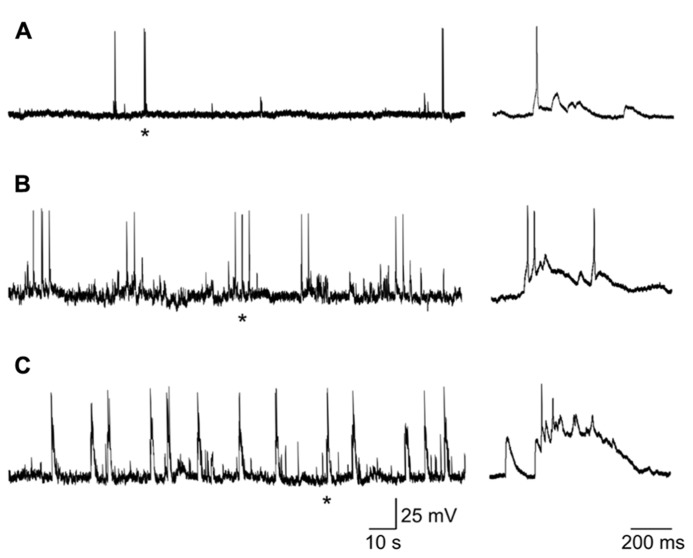
**Different patterns of coherent activities in neonatal GCs. (A)** neuron (at P1) exhibiting sporadic depolarizing synaptic potentials that in few cases reached the threshold for action potential generation. **(B)** GDPs occurring in clusters of 2–3. **(C)** GDPs occurring at more regular intervals. Single action potentials or GDPs marked with * are shown on the right on an expanded time scale.

Most (58/63) spiking neurons, recorded from P0 to P3, exhibited patterns of coherent activity reminiscent of that found in the developing Ammon’s horn (Ben-Ari et al., 1989) and described as GDPs. As in the CA1 and CA3 hippocampal regions, GDPs were either grouped in clusters of 2–5 (**Figure 6B**) or occurred at more or less regular intervals (**Figure 6C**) at the frequency of 0.1 ± 0.3 Hz, often preceded by a barrage of synaptic events. Few spiking neurons did not exhibit GDPs but only spontaneous activity either isolated or in bursts that often reached the threshold for action potential generation (**Figure 6A**).

Giant depolarizing potentials were characterized by long-lasting recurrent membrane depolarizations (up to 30 mV in amplitude) giving rise to action potentials often grouped in bursts and separated by silent periods. GDPs were network-driven events since their frequency, but not their amplitude, was unaffected by changing the membrane potential to more depolarized or hyperpolarized values.

**FIGURE 7:**
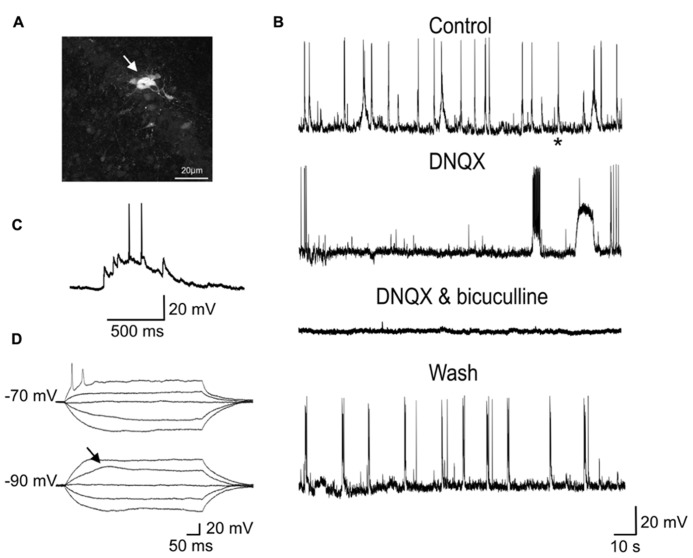
**Network-driven GDPs were reduced in frequency by DNQX and blocked by DNQX plus bicuculline. (A)** Whole-cell recording of a P2 GC with biocytin revealed a cluster of several dye-coupled neurons. **(B)** Sample traces of spontaneous activity (GDPs) recorded from the same neuron in control conditions, in DNQX (20 μM), in DNQX and bicuculline (20 μM) and after wash. In the presence of DNQX, GDPs occurred at lower frequency. In DNQX it is visible also a plateau potential. **(C)** The GDP marked with an asterisk in the control trace is shown on an expanded time scale. **(D)** Voltage responses to depolarizing and hyperpolarizing currents steps of increasing intensity obtained from the same neuron. Note sodium and low threshold calcium spikes.

Although reduced in frequency, GDPs were still present in DNQX (20 μM; **Figure 7**) suggesting that, in the absence of a glutamatergic drive, the depolarizing action of GABA was still able to exert an excitatory action at the network level. In two cases, in the presence of DNQX long-lasting (13 and 19 s duration) plateau potentials could be unveiled.

These were probably generated by the activation of intrinsic membrane conductances, known to involve small groups of neurons coupled by gap-junctions (**Figure 7**; Crépel et al., 2007). However, the rare occurrence of these events did not allow testing whether they were sensitive to gap junction uncouplers or could be blocked by hyperpolarizing the membrane toward more negative values. GDPs were completely abolished by DNQX plus bicuculline (20 μM), indicating that they were triggered by the synergistic action of both glutamate and GABA. As in immature CA3 principal cells (Ben-Ari et al., 1989), synchronized activity was blocked by TTX (1 μM) further supporting their network origin (data not shown).

## DISCUSSION

The present data clearly show that, immediately after birth, GCs in the dentate gyrus exhibit different degrees of immaturity as revealed by immunocytochemical experiments and *post hoc* morphological reconstruction of biocytin-labeled cells. Thus, at P2, only a small percentage of Prox1-positive GCs were labeled with NeuN, which is specifically activated near the end of their differentiation process (Ming and Song, 2011). In keeping with a mixed GABAergic and glutamatergic neurotransmission of immature MF (Münster-Wandowski et al., 2013), a recent study has unveiled that Prox1-positive GCs transiently express the GABA synthesizing enzyme GAD67, thus supporting the view that, immediately after birth, GCs are able to synthesize GABA in addition to glutamate (Cabezas et al., 2013). GABA released from MF terminals may activate pre (Cabezas et al., 2012) and/or postsynaptic GABA_A_ receptors (Safiulina et al., 2006, 2010) to modulate MF excitability and to generate GABA_A_ mediated postsynaptic currents in targeted neurons, respectively.

Biocytin-labeled GCs express different degrees of immaturity. In general, they revealed few dendritic branches with short dendrites barely penetrating into the molecular layer and exhibiting varicosities and filopodia. In contrast with their dendritic arborization the axons of immature GCs, the MF, were able to reach the CA3 pyramidal layer already at P1. Although we don’t know whether GCs axons made synaptic contacts with principal cells, it is likely that, similarly to the visual system (Kasper et al., 1994), the maturation of the dendritic tree takes place after GC axons have reached the CA3 subfield, supporting the view that this process is influenced by retrograde signals (Jones et al., 2003). Previous studies have shown that mature adult-like GCs, characterized by elongated dendrites (with spines) penetrating into the molecular layer, start appearing toward the end of the first postnatal week (Jones et al., 2003).

Our electrophysiological data obtained from P0 to P3 old rats unveiled passive membrane properties similar to those obtained from older rats at P5–P8 (Liu et al., 1996; Liu et al., 2000; Ye et al., 2000; Ambrogini et al., 2004), indicating that a certain degree of immaturity persists at late developmental stages. Usually, maturity is characterized by progressive more hyperpolarized values of RMP, decrease in membrane time constant, in R_in_ and increase in membrane capacitance (Spigelman et al., 1992). However, in the present case, these values were rather scattered and no significant differences were observed between GCs recorded at P0 and P3. All patched cells exhibited high values of R_in_. However, respect to more mature cells, immature neurons with rudimentary spikes displayed higher R_in_ values associated with lower capacitance. These factors, combined with the compact size of immature GCs, likely contribute to their high degree of excitability, such that even small fluctuations in membrane conductance may produce large voltage responses. This can be attributed to changes in the expression of intracellular anions and potassium efflux. Moreover, a developmentally regulated expression of voltage-gated sodium, calcium and potassium channels (Spigelman et al., 1992) may account for differences in active membrane properties such as spike amplitude and duration. The fact that rudimentary spikes were blocked by TTX suggests that voltage-dependent sodium channels are responsible for spike genesis. Interestingly, rudimentary sodium spikes were accompanied with low threshold calcium spikes. T-type Ca^2^^+^ currents underling low threshold calcium spikes have been originally described in sensory neurons where they are developmentally regulated since they disappear during the first few weeks of postnatal life, suggesting a major role in the generation of oscillatory activities (Huguenard, 1996). In the cerebellum, the developmental expression of low threshold calcium spikes parallels that of the dendritic tree, indicating a possible dendritic localization of this conductance (Gruol et al., 1992). In the present experiments we cannot exclude the involvement of calcium conductances localized on dendrites. However, this hypothesis seems unlikely since maturation of GC dendrites is usually associated with the loss of low threshold calcium spikes. Although the functional role of low threshold calcium channels in immature GCs is still unclear, these may boost calcium entry *via* high threshold calcium channels and/or NMDA receptors following the depolarizing action of GABA thus contributing to GDPs generation. The transient elevation in intracellular calcium level during GDPs activates signaling pathways known to control several developmental processes, including DNA synthesis, neuronal migration, differentiation, and synaptogenesis (Cherubini et al., 2011). It is worth mentioning that in adult-born GCs low threshold calcium currents were present only in cells with synaptic inputs, suggesting that T-type of channels may play a crucial role in cell differentiation and in synaptic plasticity processes (Ambrogini et al., 2004; Schmidt-Hieber et al., 2004).

Although the present experiments clearly show that immature GCs are in several aspects similar to adult-born neurons in the inner GC layer (Laplagne et al., 2006, 2007; Overstreet-Wadiche and Westbrook, 2006; Overstreet-Wadiche et al., 2006; Zhao et al., 2010), they are functionally different since, unlike adult-born GCs, immature GCs display network-driven GDPs. This can be attributed to the depolarizing and excitatory action of GABA that, compared with the adult hippocampus, early in postnatal life is very pronounced. GABA-induced membrane depolarization may act in synergy with glutamate to synchronize neuronal networks. GDPs have been already described in the fascia dentata of immature rabbits (Menendez de la Prida et al., 1998) and rats (Hollrigel et al., 1998). In rabbits GDPs persisted when the dentate gyrus was isolated from the Ammon’s horn indicating that the entire hippocampal network possesses the capacity to generate them.

Here, the observation that GDPs persisted at lower frequency in the presence of the AMPA/kainate receptor antagonist DNQX strongly suggests the depolarizing and excitatory action of GABA is crucial for network synchronization. In addition, in immature GCs, oscillatory activity can be facilitated by the slow kinetics of GABA_A_-mediated synaptic currents that may contribute to integrate incoming excitatory inputs (both GABAergic and glutamatergic) over a large time window (Draguhn and Heinemann, 1996; Hollrigel and Soltesz, 1997). In analogy with the synchronized activity generated in the disinhibited hippocampus (de la Prida et al., 2006), GDPs emerge when a sufficient number of cells fire and the excitability of the network attains a certain threshold within a restricted time window. Dye-coupling between immature GCs would facilitate this task.

Although neonatal and adult neurogenesis in the dentate gyrus seem to follow similar steps, the possibility that early synchronized activity early in postnatal development may play a role in synaptic wiring, thus contributing to refine local neuronal circuits according to aphorism “neurons that fire together wire together,” cannot be excluded. Therefore, it is likely that changes in the environmental factors such as activity may determine the different phenotype of neonatal or adult GCs progenitors.

## AUTHOR CONTRIBUTIONS

Andrea Pedroni, Antonello Mallamaci and Enrico Cherubini conceived and designed the experiments. Andrea Pedroni performed most of the electrophysiological and immunocytochemical experiments and analyzed the data. Do Duc Minh performed some immunocytochemical experiments. Enrico Cherubini wrote the paper with the approval of all the authors.

## Conflict of Interest Statement

The authors declare that the research was conducted in the absence of any commercial or financial relationships that could be construed as a potential conflict of interest.
